# Case Report: A rare case of gastric cardia bronchogenic cyst: diagnostic challenges and EUS findings with elevated cyst-fluid tumor markers

**DOI:** 10.3389/fmed.2026.1849801

**Published:** 2026-06-19

**Authors:** Jun Huang, Qinfang Zheng, Nan Yi, Zhao Fu, JingJing Qi, Dongliang Cen, Likun Zhong

**Affiliations:** 1 Department of Gastroenterology, The People's Hospital of Guangxi Zhuang Autonomous Region, Nanning, China; 2 Department of Radiology, The People's Hospital of Guangxi Zhuang Autonomous Region, Nanning, China; 3 Department of Pathology, The People's Hospital of Guangxi Zhuang Autonomous Region, Nanning, China

**Keywords:** bronchogenic cysts, case report, cyst-fluid analysis, diagnosis, endoscopic ultrasound, gastric cardia

## Abstract

Bronchogenic cysts are rare congenital foregut malformations that most commonly occur in the thoracic cavity, whereas involvement of the gastric cardia is exceptionally uncommon. We report the case of a 36-year-old man who presented with abdominal distension and was found to have a cystic lesion adjacent to the gastric cardia. Contrast-enhanced computed tomography and magnetic resonance imaging demonstrated a well-circumscribed, non-enhancing cystic mass, but the exact origin and nature of the lesion remained uncertain. Endoscopic ultrasound showed a 4.5 × 5.0 cm homogeneous hypoechoic lesion arising from the muscularis propria layer of the gastric cardia. Endoscopic ultrasound-guided fine-needle aspiration yielded dark-brown, turbid cyst fluid, and cyst-fluid analysis showed a neutrophil-predominant, exudative profile with markedly elevated tumor-associated markers, including CEA, CA19-9, CA125, and CYFRA 21-1. Microbiological smear and culture examinations for bacteria, fungi, and mycobacteria were negative. Given the patient's symptoms, lesion size, uncertain diagnosis, abnormal cyst-fluid profile, and inability to exclude a cystic neoplasm preoperatively, laparoscopic cyst excision with gastric wall repair was performed. Histopathological examination confirmed the diagnosis of a bronchogenic cyst, characterized by pseudostratified ciliated columnar epithelium and smooth muscle within the cyst wall. The patient recovered uneventfully, and no recurrence was observed during 1 year of follow-up. This case highlights the diagnostic challenges of gastric bronchogenic cysts and the complementary role of endoscopic ultrasound in localizing and characterizing subepithelial cystic lesions. Elevated cyst-fluid tumor markers should be interpreted cautiously and should not be regarded as evidence of malignancy in isolation, as their diagnostic and prognostic significance in gastric bronchogenic cysts remains uncertain.

## Introduction

Bronchogenic cysts (BCs) are rare congenital benign foregut malformations, with an estimated prevalence of approximately 1 in 42,000 to 1 in 68,000 individuals (1). BCs can be classified into three types: mediastinal, intrapulmonary, and ectopic, with the ectopic type being the rarest. Gastric BC is an extremely uncommon ectopic foregut cyst, and its preoperative recognition remains challenging because it may mimic gastrointestinal stromal tumor (GIST), gastric leiomyoma, duplication cyst, pancreatic cystic lesion, or extrinsic compression. In this report, we describe a gastric cardia BC characterized by EUS and cyst-fluid analysis. The focus of this report is not to claim diagnostic certainty from cyst-fluid tumor markers, but to present them as unusual observations, analyze differential diagnosis and management, and clarify the limitations of interpreting these markers from a single case.

The report is structured according to the CARE framework (2), with additional details on patient information, diagnostic reasoning, therapeutic decision-making, follow-up, patient perspective, and a timeline in Table 1.

**Table 1:** Timeline of the episode of care.

Time point	Clinical event	Key findings/management
>1 month before admission	Symptom onset	Abdominal distension without other documented systemic symptoms.
6 days before admission	Gastric cardia mass detected	A cystic mass near the gastric cardia was identified during imaging evaluation.
Admission	Initial assessment	No obvious abnormality on physical examination or routine laboratory tests.
Diagnostic work-up	CT/MRI and gastroscopy	Non-enhancing cystic lesion adjacent to the gastric cardia; subepithelial protrusion with intact but congested mucosa.
EUS-FNA	Endosonographic characterization and aspiration	4.5 × 5.0 cm hypoechoic lesion apparently from muscularis propria; dark-brown, turbid cyst fluid; markedly elevated nucleated cell count with polymorphonuclear leukocyte predominance and elevated tumor markers.
Preoperative decision	Multidisciplinary discussion	Surgery selected rather than surveillance or repeat aspiration because of symptoms, lesion size, uncertain histology, apparent muscularis propria involvement, and abnormal dark-brown/turbid cyst-fluid findings; elevated markers contributed only to diagnostic uncertainty.
Operation	Laparoscopic exploration and resection	Abdominal cyst resection, gastric wall repair, and abdominal drainage.
Postoperative pathology	Definitive diagnosis	Pseudostratified ciliated columnar epithelium and cyst wall with fibrous tissue, smooth muscle, adipose tissue, calcifications, cholesterol crystals, and foamy histiocytes; no malignancy.
12-month follow-up	Outcome assessment	No abdominal distension or discomfort; follow-up imaging showed no recurrent abdominal mass.

## Case presentation

### Patient information and clinical findings

A 36-year-old male patient was admitted to the hospital with a chief complaint of abdominal distension lasting for more than 1 month and detection of a gastric cardia mass 6 days before admission. He had no significant past medical history, and no previous related interventions were documented. No relevant family history, genetic history, or psychosocial factors related to the current disease were recorded. On admission, physical examination and routine laboratory tests revealed no obvious abnormality.

### Diagnostic assessment

Abdominal contrast-enhanced computed tomography (CT) demonstrated a rounded, well-circumscribed, fluid-attenuating lesion along the gastric cardia. A thin, uniform peripheral wall was observed, and the demarcation from the gastric wall was indistinct. The lesion contents showed no enhancement, supporting a cystic rather than vascular solid lesion (Figures 1A–C). Magnetic resonance imaging (MRI) showed intrinsic high signal intensity on non-contrast T1-weighted images (Figure 2A) and homogeneous high signal intensity on T2-weighted images (Figure 2B). Axial and coronal contrast-enhanced T1-weighted images showed no internal enhancement (Figures 2C, D), consistent with proteinaceous or hemorrhagic cystic content rather than an enhancing solid neoplasm.

**Figure 1:**
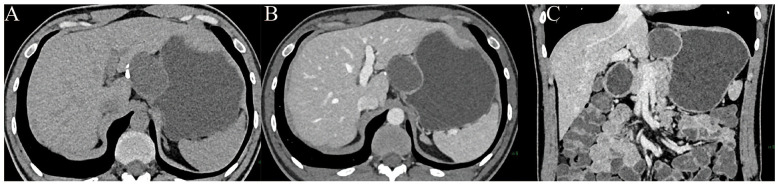
CT findings of the bronchogenic cyst. **(A)** Non-contrast CT shows a well-circumscribed, round, homogeneously hypodense lesion adjacent to the gastric cardia along the lesser curvature of the stomach. **(B, C)** Contrast-enhanced CT demonstrates no appreciable internal enhancement, supporting its cystic nature. The lesion has a thin, uniform wall and an indistinct interface with the adjacent gastric wall.

**Figure 2:**
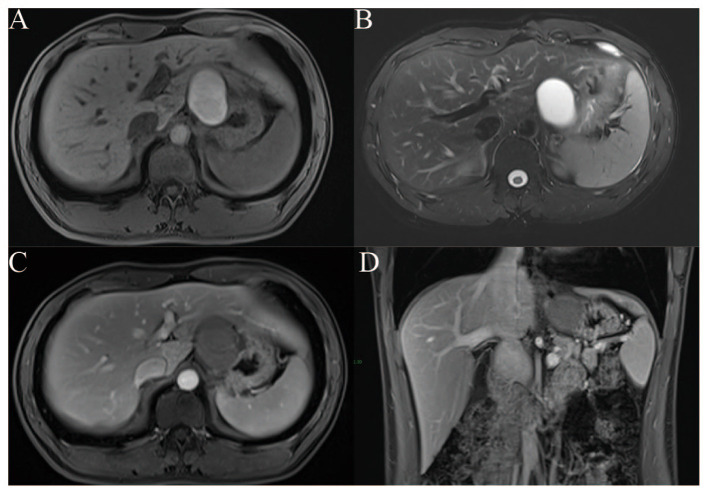
Magnetic resonance imaging (MRI) characteristics of the bronchogenic cyst. **(A)** Non-contrast T1-weighted image shows intrinsic high signal intensity, suggestive of proteinaceous or hemorrhagic cyst contents. **(B)** T2-weighted image reveals homogeneous high signal intensity, consistent with a fluid-filled cystic lesion. **(C, D)** Contrast-enhanced T1-weighted images in axial and coronal planes demonstrate no appreciable internal enhancement, supporting a cystic lesion rather than an enhancing solid neoplasm.

Gastroscopy revealed a subepithelial protrusion at the gastric cardia with intact but congested overlying mucosa (Figure 3A). EUS demonstrated a homogeneous hypoechoic lesion measuring approximately 4.5 × 5.0 cm, with well-defined margins and apparent origin from the muscularis propria layer (Figures 3B, C). Because the lesion was larger than 20 mm, symptomatic, and diagnostically indeterminate, EUS-FNA was performed using a Cook 19-G needle. Dark-brown, markedly turbid cyst fluid was aspirated (Figure 3D).

**Figure 3:**
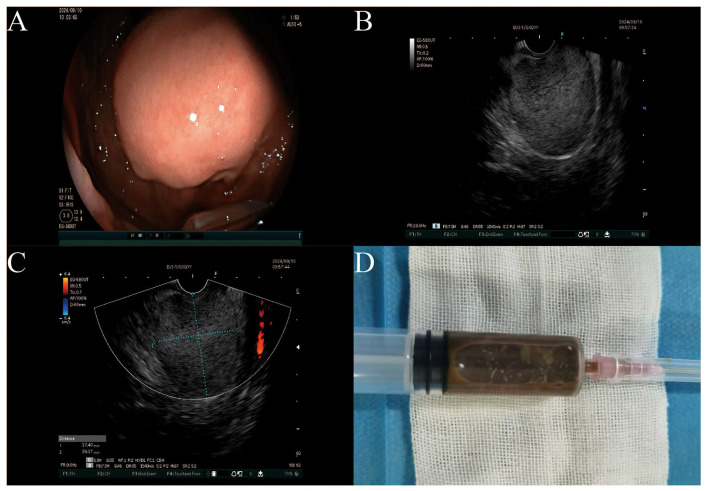
Endoscopic and endosonographic findings. **(A)** Gastroscopy reveals a subepithelial protrusion at the gastric cardia with intact but congested overlying mucosa. **(B, C)** EUS shows a homogeneous hypoechoic lesion measuring 4.5 × 5.0 cm, with well-defined margins and an apparent origin from the muscularis propria layer. **(D)** EUS-FNA yielded dark-brown, turbid cyst fluid.

Cyst-fluid analysis revealed a nucleated cell count of 89,388 × 10^6^/L with polymorphonuclear leukocyte predominance (92%) and a positive Rivalta test, indicating a neutrophil-predominant, exudative cyst-fluid profile. Microbiological evaluation was performed at the same time: bacterial, fungal, and mycobacterial smear and culture examinations were negative, whereas cytological evaluation of the aspirate was not documented. Tumor marker analysis showed markedly elevated levels of CYFRA 21-1 (cytokeratin 19 fragment) (>500.00 ng/mL), CA19-9 (>1,000.00 U/mL), CA125 (3,413.00 U/mL), CA72-4 (105.00 U/mL), squamous cell carcinoma antigen (SCCA) (>70.00 ng/mL), and CEA (>1,000.00 ng/mL).

Before surgery, the main differential diagnoses included GIST with cystic degeneration, gastric leiomyoma, gastric duplication/foregut cyst, pancreatic cystic lesion, ectopic pancreas with cystic change, vascular lesion, extrinsic compression, and ectopic BC. The EUS-based differential diagnosis is summarized in Table 2. Although the smooth margin, lack of enhancement, and homogeneous cystic appearance favored a benign cystic lesion, the large size, apparent muscularis propria origin, abnormal neutrophil-predominant exudative cyst-fluid profile, and markedly elevated cyst-fluid tumor markers left clinically important uncertainty before resection.

**Table 2:** EUS-based differential diagnosis of a gastric cardia cystic/submucosal lesion.

Differential diagnosis	Typical EUS/imaging clues	Features supporting consideration in this case	Features arguing against or limiting the diagnosis
GIST with cystic degeneration	Usually hypoechoic lesion from muscularis propria; may show irregular margins, heterogeneous echogenicity, necrosis/cystic spaces, and enhancement of solid components.	Muscularis propria origin and size >20 mm made GIST an important preoperative consideration.	CT/MRI showed no internal enhancement and EUS suggested homogeneous cystic content; pathology confirmed BC rather than GIST.
Gastric leiomyoma	Homogeneous hypoechoic lesion, usually from muscularis propria; typically solid and well circumscribed.	Well-defined hypoechoic lesion from muscularis propria.	Large fluid-filled lesion and dark-brown, turbid cyst fluid were atypical; pathology excluded leiomyoma.
Gastric duplication/foregut cyst	Anechoic or hypoechoic cystic lesion with smooth wall; may show layered wall and close relationship to gastric wall.	Non-enhancing cystic lesion with smooth margins and gastric wall continuity.	Histology showed respiratory-type ciliated epithelium compatible with BC, an ectopic foregut cyst subtype.
Extrinsic compression from adjacent lesions	Subepithelial-like bulge with an intact gastric wall layer pattern; EUS may show a lesion outside the gastric wall rather than arising from a specific wall layer.	CT/MRI showed a perigastric cystic mass and an indistinct interface with the gastric wall.	EUS favored an apparent muscularis propria origin, and surgical pathology confirmed a bronchogenic cyst associated with the gastric wall.
Ectopic pancreas with cystic change	Heterogeneous subepithelial lesion; may contain duct-like structures; usually antral but can occur elsewhere.	Subepithelial appearance can mimic other gastric SELs.	Cardia location and purely cystic features without duct-like structures made this less likely.
Pancreatic pseudocyst or mucinous pancreatic cystic lesion	Usually related to pancreas; may show septations, debris, mural nodule, or communication with pancreas/ductal system.	Proteinaceous/turbid cyst fluid and tumor marker elevation raised concern for a cystic lesion.	No documented pancreatitis; EUS favored gastric wall/muscularis propria origin rather than pancreatic origin.
Vascular lesion/varix	Anechoic serpiginous lesion with Doppler flow; enhancement may be present.	Cystic or anechoic appearance can be misleading if vascularity is not assessed.	No enhancement on CT/MRI and non-bloody cyst contents argued against a vascular lesion.
Bronchogenic cyst	Ectopic foregut cyst; EUS may show anechoic/hypoechoic cystic lesion with mucoid debris or punctate echogenic foci.	Cardia/fundus predilection, cystic nature, proteinaceous/turbid fluid, and final histology supported BC.	Preoperative features are nonspecific and overlap with other foregut cysts and cystic neoplasms.

### Therapeutic intervention

After discussion among gastroenterology, radiology, and surgical teams and after management options were explained to the patient, laparoscopic resection was selected, and the patient provided consent for surgery. The decision was based on the symptomatic presentation, lesion size of approximately 5 cm, muscularis propria involvement, uncertain preoperative diagnosis, abnormal cyst-fluid profile (dark-brown turbidity with marked leukocytosis), and need to exclude GIST or another cystic neoplasm. Surveillance was considered less appropriate because the lesion was symptomatic and larger than 20 mm. Repeat sampling was not favored because the first aspiration had already yielded abnormal cyst-fluid findings, the preoperative diagnosis remained uncertain, and additional puncture might increase procedure-related infection or leakage risk without ensuring a definitive diagnosis. The patient underwent laparoscopic exploration, abdominal cyst excision, partial gastric wall repair, and abdominal drainage tube placement.

Histopathological examination of the resected specimen (H&E staining, 40×) showed that the cyst wall was focally lined by pseudostratified ciliated columnar epithelium and composed of fibrous tissue, smooth muscle, and adipose tissue, with focal calcification, cholesterol crystal deposition, and aggregates of foamy histiocytes, consistent with BC (Figure 4). No histological evidence of malignancy, cellular atypia, or suppurative infection was documented.

**Figure 4:**
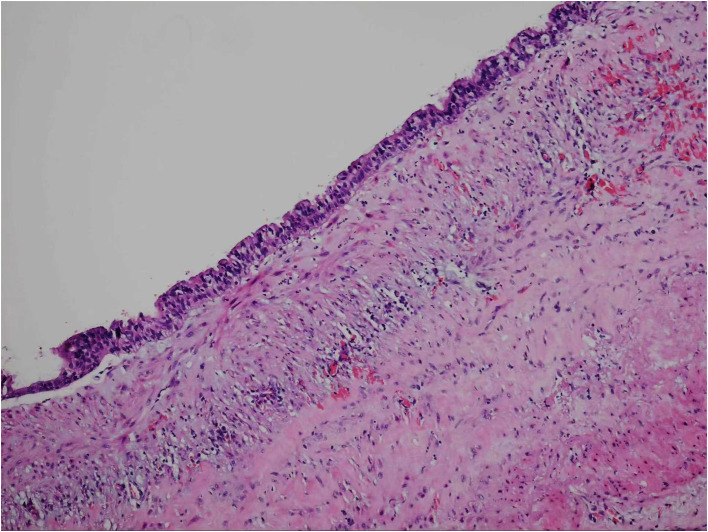
Histopathological examination of the resected specimen (H&E staining, 40 × ). The cyst wall was focally lined by pseudostratified ciliated columnar epithelium and composed of fibrous tissue, smooth muscle, and adipose tissue, with focal calcification, cholesterol crystal deposition, and aggregates of foamy histiocytes. No malignant features were identified.

### Follow-up and outcomes

The postoperative course was uneventful in the available record, and no procedure-related adverse event was documented. The patient reported resolution of abdominal distension and no abdominal discomfort after surgery. Follow-up imaging at 1 year revealed no mass formation or recurrence in the abdominal cavity. No additional oncological or antimicrobial treatment was documented after definitive pathological diagnosis.

### Patient perspective

A structured written patient-perspective statement was not prospectively collected, which is a limitation of this case report. During postoperative follow-up, the patient reported no abdominal distension or discomfort and accepted the surgical management after explanation that pathology confirmed a benign bronchogenic cyst.

## Discussion

Gastric submucosal or protruding lesions are commonly associated with GISTs, gastric leiomyomas, and gastric polyps. Other potential etiologies include ectopic pancreas, duplication cyst, pancreatic cystic lesion, vascular lesion, and extrinsic compression. In diagnostically ambiguous cases, EUS provides critical complementary value to cross-sectional imaging by delineating the lesion's layer of origin, internal architecture, vascularity, and cystic-solid differentiation. Current guidance for gastrointestinal submucosal lesions emphasizes EUS as an important tool for characterizing size, location, layer of origin, echogenicity, and shape, while also recognizing that EUS alone cannot distinguish all SEL types (3–5). In the present case, CT/MRI identified a non-enhancing cystic lesion but could not confirm the etiology. EUS localized the lesion to the muscularis propria and demonstrated a homogeneous hypoechoic cystic pattern with smooth margins. These findings narrowed, but did not eliminate, the differential diagnosis; therefore, management required correlation with symptoms, lesion size, cyst-fluid appearance, and pathological confirmation.

BC is a rare congenital condition resulting from abnormal development of the bronchial system during the embryonic period. It occurs when aberrant bronchial buds separate from the bronchial tree and persist in ectopic locations. The accumulation and expansion of secretory products within these ectopic sites, due to the inability to drain, ultimately lead to cyst formation (6, 7). Most BCs are located within the thoracic cavity, typically around the bronchi. In rare cases, abnormal bronchial buds may detach and migrate, resulting in cyst formation in locations such as the pericardium, esophagus, retroperitoneum, or stomach (8). Gastric BCs are uncommon and are most frequently found in the gastric fundus or cardia (9), consistent with the present case. Clinical manifestations are non-specific, although large cysts may cause compression-related symptoms such as abdominal distension, epigastric discomfort, nausea, or belching.

Due to the lack of specific laboratory findings and imaging characteristics, gastric BC is often discovered incidentally during routine health examinations or during evaluation for non-specific symptoms. Despite advances in imaging, a definitive preoperative diagnosis remains challenging. CT or MRI may reveal the cystic nature of the lesion; however, if BC is complicated by infection, hemorrhage, or proteinaceous/calcareous content, its density or signal may mimic a solid tumor (10). Consequently, reliance on history, clinical signs, laboratory tests, or cross-sectional imaging alone is insufficient for conclusive diagnosis, and misdiagnosis as GIST or another SEL has been repeatedly reported (11).

EUS is useful for differentiating cystic from solid masses and for determining layer of origin and relationship to the gastric wall. However, this case also illustrates the limits of EUS. Abdominal BCs are heterogeneous in location and internal appearance. Reported cases describe hypoechoic or anechoic cystic lesions, sometimes with mucoid debris or punctate hyperechoic foci. For example, peripancreatic or pancreatic-tail BCs have been reported with internal hyperechoic spots, the so-called “starry sky” appearance, mural nodules, or calcification (12, 13). Therefore, EUS findings should be interpreted as part of a broader diagnostic pathway rather than as pathognomonic evidence.

The safety and indication for EUS-FNA also require careful consideration. Potential risks include procedure-related infection, bleeding, perforation, cyst leakage or rupture, and, for suspected neoplastic cysts, theoretical tumor seeding. Evidence from pancreatic cystic lesions suggests that post-FNA infection is uncommon and that the value of routine antibiotic prophylaxis remains debated, but these data cannot be directly extrapolated to gastric BC because of its rarity and different anatomy (14, 15). In the present case, FNA was considered reasonable because the lesion was symptomatic, large, and diagnostically indeterminate. The dark-brown, turbid aspirate with marked leukocytosis also illustrates the value of obtaining microbiological and cytological studies when feasible; the negative bacterial, fungal, and mycobacterial smear/culture results argued against proven infection, although they did not by themselves explain the neutrophil-predominant profile.

Cyst-fluid tumor markers should be interpreted cautiously. Elevated tumor markers in BC are not unique to the present case. High CA19-9 has been reported in thoracic BC fluid and serum (16), and elevated tumor markers in gastric BC fluid have also been described in previous case reports (17–19). Thus, rather than representing a first report of elevated cyst-fluid tumor markers, this case adds a gastric cardia BC with detailed EUS features, an abnormal neutrophil-predominant/exudative cyst-fluid profile, and broad tumor-marker elevation. Selected relevant reports are summarized in Table 3.

**Table 3:** Selected literature relevant to bronchogenic cysts, EUS assessment, and cyst-fluid tumor markers.

Reference	Lesion/site	Preoperative diagnostic approach	Cyst-fluid/serum tumor-marker information	Relevance to the present case
Tokuchi et al. (16)	Thoracic BC	Imaging and surgical pathology	Markedly elevated CA19-9 in cyst fluid and serum; pathology benign.	Demonstrates that tumor-marker elevation can occur in benign BC and should not automatically imply malignancy.
Xiao et al. (11)	Gastric cardia ectopic BC	Preoperatively considered GIST	No detailed cyst-fluid marker profile reported in the cited manuscript.	Shows frequent preoperative misdiagnosis of gastric cardia BC as GIST.
Cassiani et al. (12)	Abdominal/peripancreatic BC	EUS through-the-needle biopsy	Main contribution was EUS-guided tissue diagnosis rather than broad tumor-marker analysis.	Supports the value of EUS-based sampling in selected abdominal BCs.
Kouanda et al. (13)	Pancreatic-tail BC	EUS-guided microforceps biopsy	Marker data not central; EUS showed a cystic lesion with internal hyperechoic foci.	Illustrates variability of abdominal BC EUS appearances.
Duan et al. (17)	Gastric BC	Surgical/pathological diagnosis	Reported elevated tumor markers in cyst contents.	Shows that elevated tumor markers in gastric BC fluid have been previously reported.
Qian and Xu (18)	Gastric fundus BC	Surgical/pathological diagnosis	Reported elevated CA72-4.	Further demonstrates that isolated or selected tumor-marker elevation has been described in gastric BC.
Chen et al. (19)	Posterior wall of gastric fundus BC	Surgical/pathological diagnosis	Reported elevated CEA and CA19-9 in cyst fluid.	Provides a recent example of gastric BC with elevated cyst-fluid markers, supporting the need to avoid unsupported priority claims.
Present case	Gastric cardia BC	CT/MRI, gastroscopy, EUS-FNA, and surgical pathology	Neutrophil-predominant/exudative cyst-fluid profile plus broad tumor-marker elevation: CYFRA 21-1, CA19-9, CA125, CA72-4, SCCA, and CEA.	Adds detailed EUS features and emphasizes that marker elevation is hypothesis-generating rather than diagnostic.

Several speculative explanations may account for the elevated cyst-fluid markers in this case, but none can be proven from a single observation. First, respiratory-type epithelium or metaplastic foregut epithelium may secrete mucin-associated antigens such as CA19-9 or CEA. Second, the neutrophil-predominant/exudative cyst-fluid profile may reflect non-specific cellular changes within long-standing cyst contents; this interpretation should be considered in light of the negative microbiological smear and culture results. Third, long-standing cyst contents, cholesterol crystals, hemorrhagic/proteinaceous debris, or necrotic material could concentrate analytes and interfere with immunoassays. Importantly, the resected specimen showed no cellular atypia, malignant histopathology, or documented suppurative infection. Therefore, the tumor-marker elevations should be regarded as non-specific and investigational, not as evidence of malignant transformation or as an independent indication for surgery.

The indication for surgery in this case should be understood within risk stratification rather than as a response to tumor markers alone. No disease-specific management guideline exists for gastric BC; therefore, clinical decisions should be individualized. Current expert guidance for gastric subepithelial lesions supports EUS-based evaluation and tissue acquisition for lesions arising from the muscularis propria, particularly when lesions are larger than 20 mm, symptomatic, or diagnostically uncertain (3–5). This patient had abdominal distension, a 4.5 × 5.0 cm lesion, apparent muscularis propria involvement, uncertain histology, and an abnormal neutrophil-predominant/exudative cyst-fluid profile. These factors collectively made definitive resection reasonable. If a smaller, asymptomatic, confidently benign cystic lesion were encountered, surveillance or repeat targeted sampling could be considered instead after individualized discussion with the patient.

This report has several limitations. It is a single case, so no causal or prognostic inference can be made. Cytological evaluation of the aspirate was not documented, limiting assessment of cellular atypia in the fluid. Although microbiological smear and culture examinations were negative as reported above, these results do not fully define the cause of the marked neutrophil-predominant/exudative cyst-fluid profile. Serum tumor-marker levels were not systematically correlated with cyst-fluid markers, and immunohistochemical staining for marker expression in the cyst epithelium was not performed. Follow-up was limited to 1 year. Future multicenter case registries should correlate EUS morphology, cyst-fluid biochemistry, microbiology, cytology, histopathology, and long-term outcomes to determine whether any cyst-fluid marker has diagnostic or prognostic value in ectopic foregut cysts.

In conclusion, gastric cardia BC is rare and remains difficult to diagnose preoperatively. EUS can define the layer of origin and cystic architecture, and selective cyst-fluid analysis may provide supportive information when interpreted with imaging and clinical context. However, elevated cyst-fluid tumor markers should be interpreted cautiously and should not be used alone to infer malignancy. In symptomatic, large, diagnostically uncertain lesions, multidisciplinary discussion and surgical resection remain reasonable for definitive diagnosis and treatment.

## Data Availability

The raw data supporting the conclusions of this article will be made available by the authors, without undue reservation.
